# Automatic Processing Advantage of Cartoon Face in Internet Gaming Disorder: Evidence From P100, N170, P200, and MMN

**DOI:** 10.3389/fpsyt.2019.00824

**Published:** 2019-11-08

**Authors:** Jinbo He, Yang Zheng, Liyan Fan, Ting Pan, Yufeng Nie

**Affiliations:** Key Laboratory of Adolescent Cyberpsychology and Behavior of Ministry of Education, Key Laboratory of Human Development and Mental Health of Hubei Province, School of Psychology, Central China Normal University, Wuhan, China

**Keywords:** Internet gaming disorder, face processing, automatic processing, feature detection, event-related potentials

## Abstract

Individuals with Internet gaming disorder (IGD) show deficits in face processing due to long-term Internet-game social activities based on cartoon faces in the popular online game "Strike of Kings." However, the abnormal neurocognitive mechanism of face recognition and processing in individuals with IGD has not been systematically explored. This study used event-related potential (ERP) methods and the reversed deviant-standard oddball paradigm to comprehensively compare four ERP components, namely, P100, N170, P200, and mismatch negativity (MMN), induced in the unconscious and automatic processing of realistic and cartoon faces in individuals with IGD. Results showed that, with respect to cartoon faces, individuals with IGD exhibited not only P100, P200 and MMN enhancements but also the absence of the N170 dominance effect in the left hemisphere. Our results also demonstrated that individuals with IGD had the advantages of early automatic perception of cartoon faces and automatic detection of changes in "cartoon" features. This study enhances our understanding of the mechanism of IGD from the neurocognitive perspective and provides candidate electrophysiological indicators for the clinical diagnosis of IGD.

## Introduction

The number of teenagers addicted to Internet games is increasing, and this addiction causes serious damage to their physical and mental health (1–4). The new version of DSM-5 (5) lists Internet gaming disorder (IGD) as one of the mental disorders to be studied. The newly released ICD-11 (6) also lists IGD as a new addiction subtype. Therefore, IGD has become an urgent concern in current psychological and psychiatric medical research.

A remarkable behavioral characteristic among individuals with IGD is a social pattern characterized by increased interest in social interaction *via* Internet games and tendency to avoid necessary real-life social interactions (7–9). Face recognition and processing is the cognitive-neural basis of social interaction. The social interaction of individuals with IGD *via* Internet games relies on the processing of cartoon faces in online games, such as "Strike of Kings," a massively multiplayer online role-playing game, and their addiction to Internet games reduces their real-life communication, which requires the processing of realistic faces (10). If this situation occurs in the long term, individuals with IGD increasingly communicate based on cartoon faces, whereas their interaction with realistic faces decreases. The negative effect of this deviant social pattern on the face recognition and processing abilities of individuals with IGD remains to be explored.

Research on the face processing of individuals with IGD is mainly focused on the event-related potential (ERP) component N170, which reflects the specific sensitivity of faces (11, 12). N170 amplitudes elicited by faces are larger than those elicited by other objects; this phenomenon is known as the N170 face effect (13, 14). Zhao and Gao (15) investigated the neurocognitive characteristics of individuals with IGD in processing cartoon faces. They compared the amplitude difference between subjects with IGD and controls in Nd170. Specific face component Nd170 obtains by subtracting object N170 from face N170 and can reflect humans' sensitivity to faces. The results showed that the Nd170 amplitudes of individuals with IGD were significantly larger than those of controls and may thus have acquired increased neural sensitivity to cartoon faces. He et al. (16) adopted the same experimental approach and investigated the neurocognitive characteristics of realistic face processing in individuals with IGD. They found that, with respect to the processing of realistic faces, the Nd170 amplitudes of subjects with IGD were significantly smaller than those of controls. This finding demonstrated that subjects with IGD had decreased sensitivity to realistic faces. Lei et al. (10) employed the behavioral paradigm of visual search to study the attention processing of two types of expressions (positive and negative) by individuals with IGD on realistic and cartoon faces. They found that individuals with IGD show an advantage of attentional orienting with regards to cartoon faces of negative expression, suggesting that individuals with IGD can rapidly capture cartoon faces. The above studies indicated that individuals with IGD have reduced sensitivity to realistic faces and increased sensitivity to cartoon faces.

The face recognition and processing functions of individuals with IGD are suggested to be abnormal at the neurocognitive level. However, we believe that studies on these functions are not sufficiently comprehensive and intensive, basing on the following four reasons:

First, previous studies were only concerned on the abnormality of the face-specific N170 component in individuals with IGD and failed to investigate the P100 component, which reflects the processing of early perception (17), and the P200 component, which is usually linked to deeper processing of a face and is also sensitive to own-group effects (18, 19). In terms of the cognitive processing of face recognition, the early perception (P100) of the face material should exist before the processing of whether or not a face material is human (N170). Studies on substance addiction and other mental disorders have found that the ability of patients for facial processing is abnormal at the early perception stage. Maurage et al. (20) found that alcohol addicts had longer P100 latency than healthy controls when processing realistic faces. Earls et al. (21) found that subjects with schizophrenia elicit weaker P100 of realistic faces than healthy subjects. Given that IGD shares many neurocognitive features with substance addiction and mental disorder (22), these studies suggested that abnormalities in face processing in individuals with IGD may have occurred at an earlier stage of visual processing. In addition, cartoon faces have become addiction-related cues for individuals with IGD due to the long-term exposure to cartoon faces. Addicts have an advantage of early automatic perception in response to addiction-related cues, which is manifested by the increased P100 amplitude of such cues (23, 24). Therefore, individuals with IGD may have an advantage of early automatic perception to cartoon faces, resulting in enhanced P100 peak amplitude.

Similarly, there is another facial information processing between the perception and the recognition of the faces, to which the P200 component is considered to be sensitive (18, 25). For example, a comparative study of the faces of different races found that the same-race faces evoked a larger amplitude of P200 than other-race faces (26). A research of Lucas et al. (19) suggested that the amplitudes of earlier occipito-temporal P200 potentials were larger for later-remembered relative to later-forgotten other-race faces. In another study, a series of different gender faces were presented to the subjects. The results showed that sexually attractive faces evoked a larger amplitude of P200 than sexually unattractive faces, which reflected the individual's attention bias towards faces (27). For IGD individuals, the frequent occurrences of cartoon face in games have been linked with game behavior. Due to long-term contact with cartoon faces, they may be more familiar and sensitive to cartoon faces than real faces.

Second, previous studies did not continue to examine the mismatch negativity (MMN) that reflects the automatic detection of facial features (28) after face-specific processing (N170), that is, whether the face is "realistic" or "cartoon." Incentive sensitization model (29) emphasizes that long-term addictive stimulation changes the brain system function related to the reward circuit, which can gradually raise the sensitivity of the motivational center to addiction-related cues, that is, neural sensitization. Neural sensitization leads to the psychological and implicit characterization of addiction-related cues through addiction salience and causes a pathological desire for addictive behavior (30). For individuals with IGD, cartoon faces, representing online game roles, which appears repeatedly with their online behaviors, has become a cue to induce their addiction. Many studies have shown that the processing of this trigger in individuals with IGD is unconscious and automatic (31, 32). Thus, the face recognition and processing of individuals with IGD are best studied in an unconscious and automatic condition. However, the studies mentioned above were all conducted in a conscious and controlled condition. Our research adopted the oddball paradigm for unconscious and automatic processing. MMN is generally evoked by this paradigm, which reflects the automatic detection and analysis of the nervous system to changes in stimulation features in unconscious condition. The block of this paradigm is composed of standard stimuli with high probability and deviant stimuli with low probability, and the latter differ from the former in a certain feature. Given that low-probability deviant stimuli are a violation of the perceptual memory mode formed by high-probability standard stimuli, such a violation is thus named MMN. The ERPs evoked by deviant stimuli generally show a more negative wave than those evoked by standard stimuli. According to Näätänen (28), this result is largely due to the subjects' awareness of the difference between deviant and standard stimuli in a specific feature and to their automatic detection of the change in this feature. In most cases, the MMN is a difference in amplitude obtained by subtracting ERPs induced by standard stimuli from ERPs induced by the corresponding deviant stimuli (33). Cartoon and realistic faces have the same elements (e.g., face shape, eyes, nose, and mouth), but the features (e.g., cartoon and realistic feature) are different. If subjects unconsciously detect a feature change in an oddball experiment that uses realistic faces as standard stimuli with high probability and cartoon faces as deviant stimuli with low probability, they may automatically detect the "cartoon" feature of the face, and an MMN is thus produced. Conversely, if subjects unconsciously detect a feature change in an oddball experiment that uses cartoon faces as standard stimuli with high probability and realistic faces as deviant stimuli with low probability, they may automatically detect the "realistic" feature of the face, and an MMN is thus produced as well. On the basis of the relevance of cartoon faces as an addiction-related cue for individuals with IGD, we believe that individuals with IGD exhibit an advantage in automatic detection when it comes to the features of cartoon faces.

Third, previous studies did not conduct a direct comparison of the neurocognitive processing of realistic versus cartoon faces in the same experiment. Zhao et al. (15) and He et al. (16) used the same experimental paradigm but different face materials (one on cartoon faces and the other on realistic faces). As a result, the credibility of their conclusions was weakened.

Finally, the subjects with IGD recruited in previous studies were high-score subjects selected by psychology scale. These subjects were not clinically diagnosed and whether they met the clinical criteria of DSM-5 or ICD-11 could not be determined. The clinical values of these studies were thus reduced.

Therefore, we used the technology of ERP and considered the facial types (realistic face and cartoon face) as independent variables to conduct a study in which the subjects conformed to clinical diagnosis. The experiment allowed us to comprehensively investigate the neurocognitive mechanisms in the unconscious and automatic condition. We hypothesized that (1) individuals with IGD exhibit a significantly larger P100 peak amplitude when elicited by cartoon faces than when elicited by realistic faces and a significantly larger P100 peak amplitude of cartoon faces than healthy individuals; (2) individuals with IGD exhibit a significantly larger N170 peak amplitude when elicited by cartoon faces than when elicited by realistic faces and a significantly larger N170 peak amplitude of cartoon faces than healthy individuals; (3) individuals with IGD exhibit a significantly larger P200 peak amplitude when elicited by cartoon faces than when elicited by realistic faces and a significantly larger P200 peak amplitude of cartoon faces than healthy individuals; (4) the MMN amplitudes of individuals with IGD elicited by cartoon faces are significantly larger than those elicited by realistic faces, and individuals with IGD show a significantly larger MMN amplitude of cartoon faces than healthy individuals.

## Materials and Methods

### Subjects

A random sample survey of undergraduates from a university in Wuhan was conducted. The Internet addiction test (IAT) (34) and the Internet game addiction scale (IGAS) were used (35). The IAT is a psychological scale with 20 items in total, using Likert 5-point scores with a 20- to 100-score range. And the IGAS is a clinical diagnostic scale with 10 items. The participants were asked to answer "yes," scoring one point, or "no," scoring zero, and the score range was 0 to 10. A total of 400 questionnaires were distributed, and 386 valid questionnaires were collected. Through careful examination and scoring, the subjects with IGD were selected according to the following criteria: (1) the IAT score was 80 or above; (2) the IGAS score was 7 or above; (3) the type of Internet game played was mainly "Strike of Kings"; and (4) a clinical interview was administered by two psychiatrists to diagnose IGD in accordance with DSM-5. The subjects as control group were selected according to the following criteria: (1) the IAT score was 40 or below; (2) the IGAS score was 3 or below; (3) the type of Internet game played was mainly "Strike of Kings." A total of 15 male college students with "Strike of Kings" game addiction were recruited into the IGD group. And 15 non-IGD male college students were selected from the survey sample as the control group. There were no significant differences between the two groups in terms of gender, age, and profession. All subjects were right-handed and had normal or corrected-to-normal vision. Moreover, none of them had color blindness or color weakness, and neither had any history of mental illness or any medication history related to the central nervous system. They all had no history of addiction, and were asked not to use alcohol, drugs, nicotine, and other psychotropic substances within three days before the experiment. All subjects signed informed consent forms before the experimentation and received a certain amount of compensation after the experiment. This study was approved by the Ethics Committee of the School of Psychology of Central China Normal University. The subject information is shown in **Table 1**.

**Table 1:** Subject information.

	IGD (15)	Control (15)	t	p
Age	20.97 ± 1.65	21.02 ± 1.70	0.05	0.96
Daily Internet game duration (h)	6.97 ± 0.72	1.10 ± 0.54	25.25	<0.001
IAT (score)	82.13 ± 2.30	32.87 ± 2.80	51.62	<0.001
IGAS (score)	7.34 ± 0.87	2.44 ± 0.68	17.14	<0.001

IGD, Internet gaming disorder group; Control, control group; IAT, Internet addiction test; IGAS, Internet game addiction scale.

### Materials and Procedure

Fifteen cartoon faces were selected from the character library of the Internet game "Strike of Kings," and fifteen realistic faces were selected from "Chinese Facial Affective Picture System" (36). We first removed the ears, hair, and neck of all faces to avoid errors caused by the physical properties of the face. We then used Adobe Photoshop CS6 to standardize the pixel, background color, brightness, contrast, and resolution of all of the face pictures. The grayscale pictures of the face material were finally obtained. Thirty college students were randomly selected to evaluate the familiarity of the experimental materials. A five-point Likert scale was used (very familiar, 5 points; familiar, 4 points; moderately familiar, 3 points; not very familiar, 2 points; and not familiar at all, 1 point). *t*-Test was performed, and the results showed no significant difference (*t*_(29)_ = 0.75, *p* = 0.46) between cartoon faces (4.13 ± 0.74) and realistic faces (4.33 ± 0.72).

The E-Prime 2.0 software (Psychology Software Tools Inc., Sharpsburg, North Carolina, USA) was used for the experimental procedure. First, a fixation point "+" was shown at the center of the screen for 450 ms. A pair of cartoon or realistic face pictures were then presented as standard stimuli or deviant stimuli on both sides of "+." The participants were instructed to ignore the face pictures on sides of "+," to focus on "+" and to press the space bar when the fixation point "+" becomes larger or smaller as soon as possible. This procedure can induce conditions of unconscious and automatic processing (37). All subjects were given 2 min to practice before the formal experiment for them to be familiarized with the button operation. All of the face pictures were presented on the screen with an exposure duration of 150 ms, an inter-stimulus interval of 450 ms, and a visual angle of 3.68° × 3.42° from a viewing distance of 70 cm. The images were 260 pixels × 300 pixels in size and 300 dpi × 300 dpi in resolution. The screen background was white, and the pictures were dark gray.

**Figure 1:**
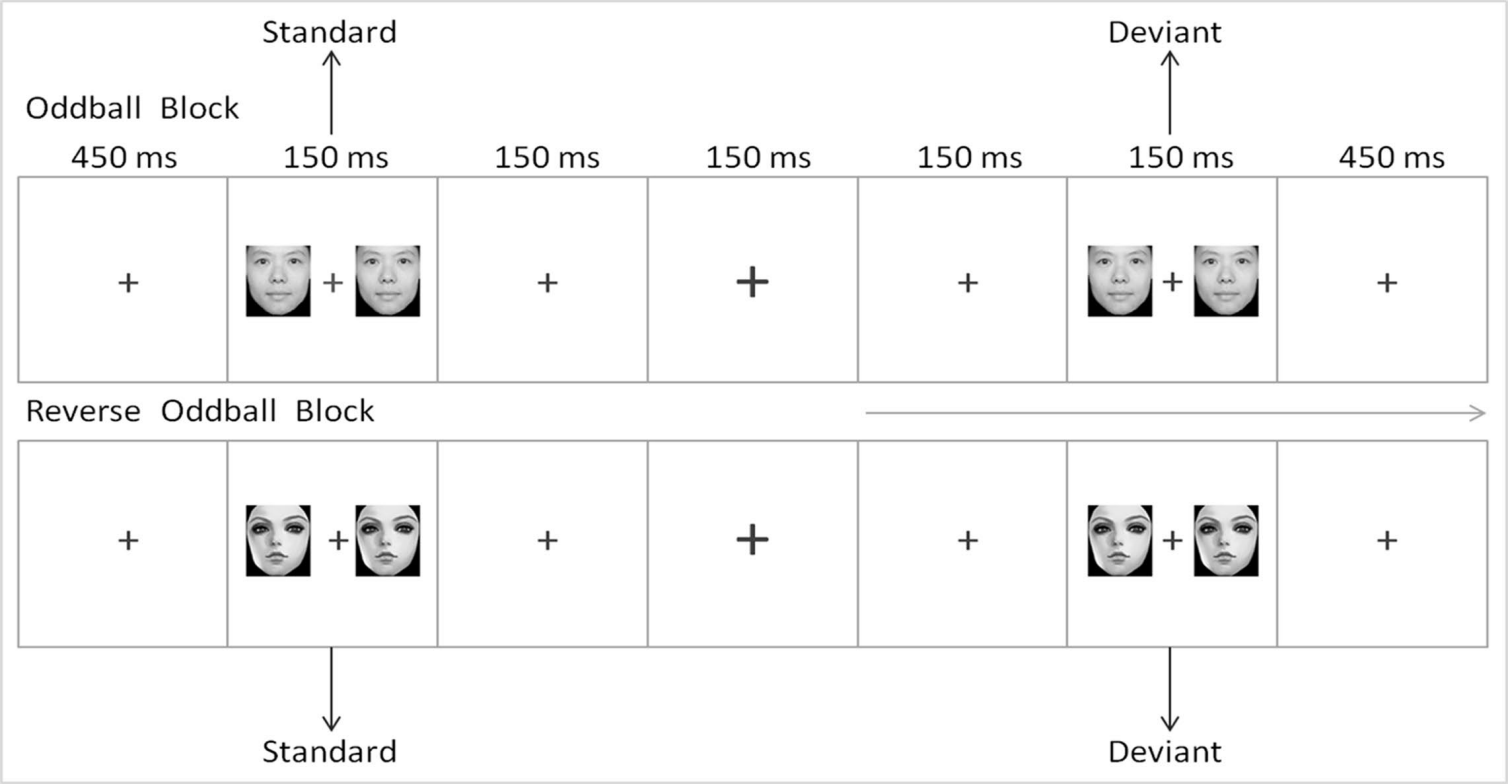
Scheme of the experimental procedure.

The reversed deviant-standard oddball paradigm was used in the experiment. Two types of face pictures (realistic and cartoon faces) were used as standard and deviant stimuli. To eliminate interference caused by the physical properties of the pictures to the MMN, we subtracted the standard ERP of the same type of face from the deviant ERP (38, 39). The experiment consisted of two blocks, each of which was composed of 480 trails. In block 1, realistic faces were used as standard stimuli, whereas cartoon faces were used as deviant stimuli. In block 2, cartoon faces were used as standard stimuli, whereas realistic faces were used as deviant stimuli. For the establishment of a sensory memory pattern, 10 standard stimuli were first presented in each block. Subsequently, the standard and deviant stimuli were presented in a pseudorandom order, and their presentation probability was 8:2. No less than two standards occurred between two deviants. The sequence of the two blocks was randomized among the subjects. The scheme of the experimental procedure is shown in **Figure 1**.

### Electrophysiological Recording and Analysis

Electroencephalogram (EEG) was continuously recorded (band pass: 0.05–100 Hz, sampling rate: 500 Hz) using a NeuroScan-64–guided EEG recording and analyzing system. The tip of the nose was used as a reference. VEOG and HEOG were recorded with two pairs of electrodes. One pair was placed above and below the right eye, and the other was placed 10 mm from the lateral canthi. All electrode impedance values were less than 5 kΩ throughout the experiment. EEG data were analyzed offline with NeuroScan (version 4.5). EOG artifacts were corrected through the method proposed by Semlitsch et al. (40). The EEG was segmented in epochs 650 ms altogether from 50 ms pre-stimulus to 600 ms post-stimulus. Peak-to-peak deflections exceeding ± 80 µV were rejected as artifacts. The averaged ERPs for each experimental condition were digitally filtered with a low-pass filter at 30 Hz (24 dB/octave).

Four types of experimental conditions (realistic-standard, realistic-deviant, cartoon-standard, and cartoon-deviant) were separately superimposed and averaged. The results showed that the P100, N170, and P200 were induced under each experimental condition and among all subjects. The ERP accepted by all subjects under each condition was higher than 60 times, and no significant difference in the number of trails accepted by ERPs among the stimulus conditions was observed. Finally, the MMNs of two groups (realistic-MMN and cartoon-MMN) were obtained by subtracting the ERPs induced by standard stimuli of pictures of the same type from those induced by deviant stimuli.

On the basis of the relevant literature (41) and the total average of the ERPs of this study, the measurement windows were selected, namely, 90–150 ms for the P100, 150–210 ms for the N170, and 210 to 270 for the P200. The average amplitudes of the MMNs were measured in two time ranges, namely, 300–400 and 400–500 ms. Visual inspection of the grand-average ERPs indicated that the trend of P100, N170, P200 and MMN waveforms in occipital and temporal occipital regions were similar. In order to investigate these four components more concentratively and intuitively, we chose PO5/PO6 as representative electrodes to present and analyze in the junctions of occipital and temporal-occipital regions.

The data were statistically analyzed using SPSS 18.0 (SPSS Inc., Chicago, IL, USA). Peak and average amplitudes were analyzed by 2 × 2 × 2 ANOVA, with participant group (IGD group, control group) as a between-subject factor and with face type (realistic, cartoon) and hemisphere (left, right) as within-subject factors. Pearson correlation coefficients were used during the correlation analyses.

## Results

### Behavioral Data

No significant difference (*t*_(29)_ = 1.11, *p* = 0.28) was found in the detection accuracy of the "+" size change between the IGD group (96.23%) and control group (97.04%). This result indicates that all of the subjects followed the experimental guidelines and focused on the changing size of "+" but ignored the pictures shown on both sides of the fixation point. Therefore, all subjects were under unconscious and automatic condition when processing picture materials.

### ERP Data

**Figure 2:**
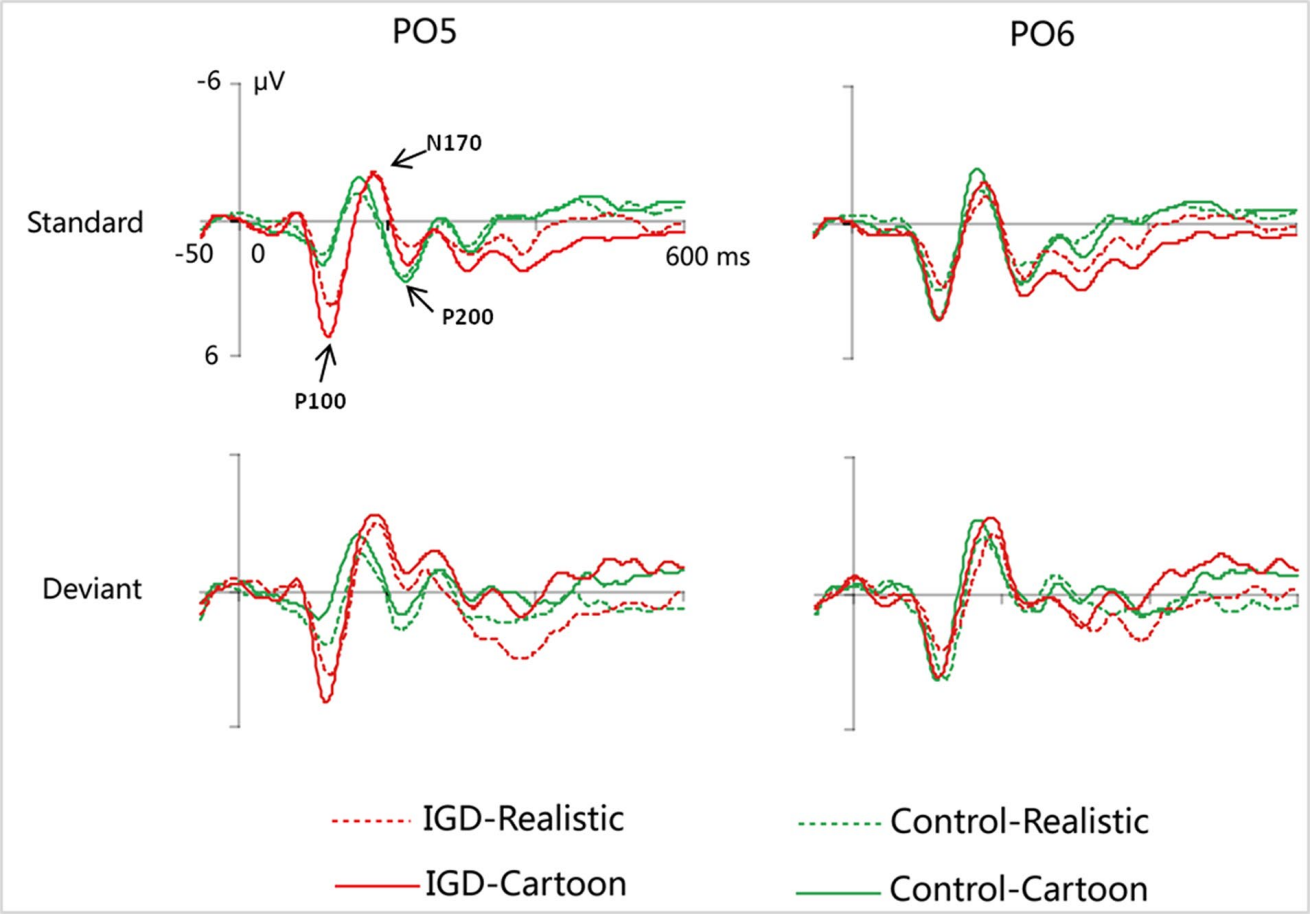
Grand-averaged P100, N170, and P200 waveforms for cartoon and realistic faces in the IGD and control groups at the PO5 and PO6 electrodes. IGD, Internet gaming disorder group; Control, control group; Standard, standard stimuli; Deviant, deviant stimuli; Realistic, realistic faces; Cartoon, cartoon faces.

**Figure 3:**
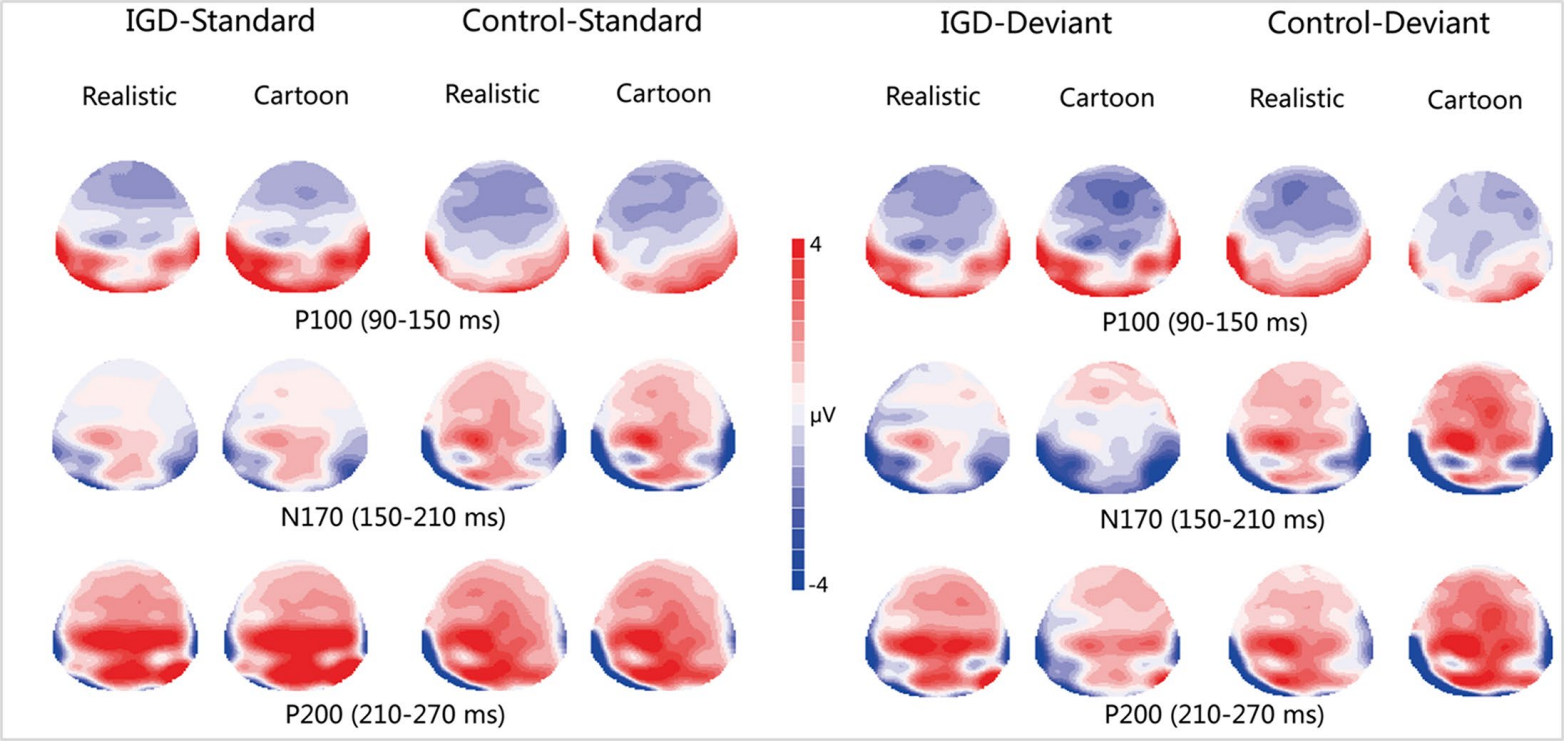
Two-dimensional topographic distribution of P100 (90–150 ms), N170 (150–210 ms), and P200 (210–270 ms) for cartoon and realistic faces in the IGD and control groups. IGD, Internet gaming disorder group; Control, control group; Standard, standard stimuli; Deviant, deviant stimuli; Realistic, realistic faces; Cartoon, cartoon faces.

**Figure 4:**
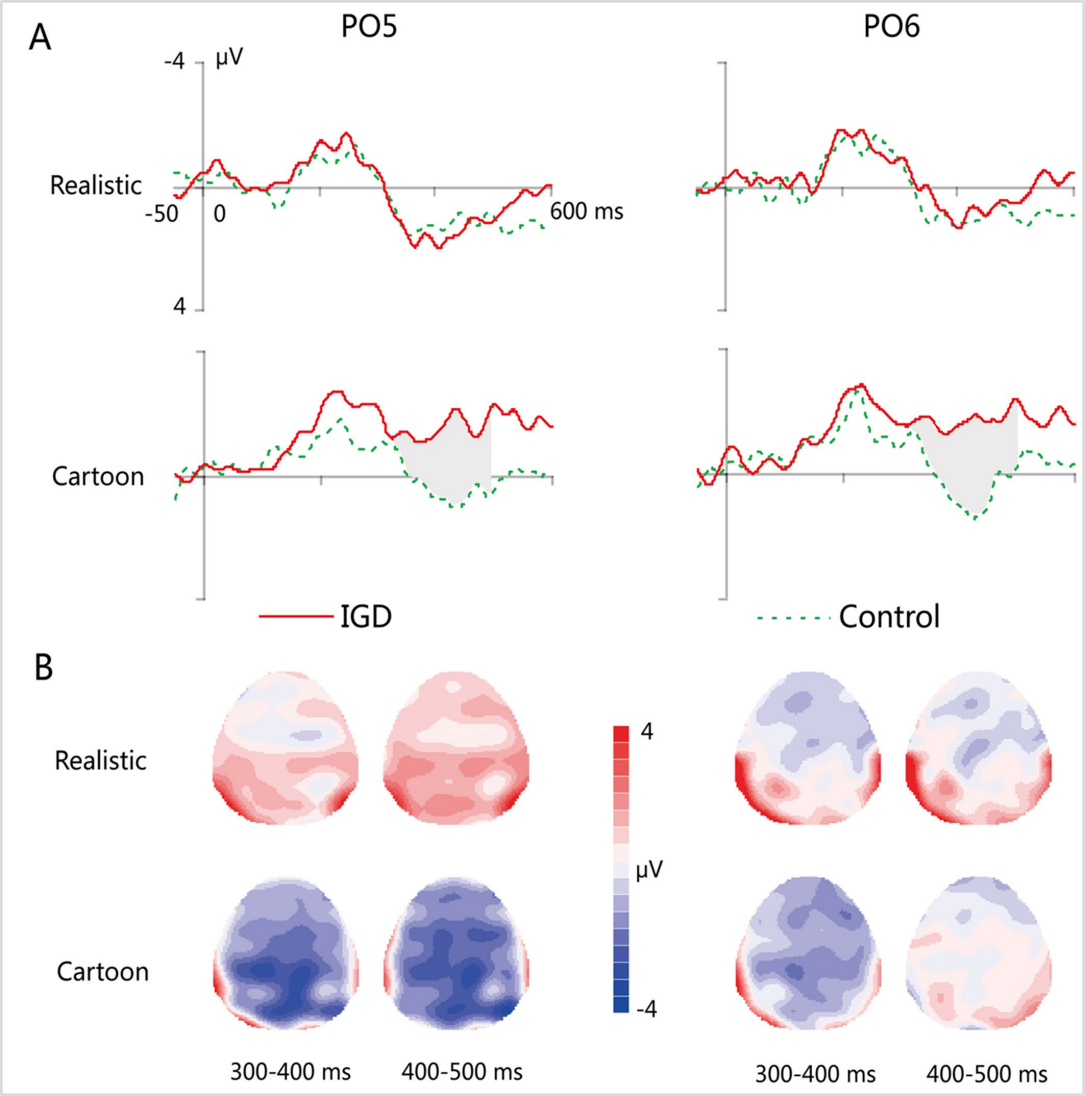
**(A)** Grand-averaged MMN waveforms for cartoon and realistic faces in the IGD and control groups at the PO5 and PO6 electrodes. **(B)** Two-dimensional topographic distribution of MMN for cartoon and realistic faces in the IGD and control groups. IGD, Internet gaming disorder group; Control, control group; Realistic, realistic faces; Cartoon, cartoon faces.

Peak amplitudes of the P100, N170 and P200 and mean amplitudes of the MMN are shown in **Tables 2**–**5**. The grand averaged ERPs are shown in **Figure 2** and a two-dimensional topography for each condition in both groups are shown in **Figure 3**. The grand averaged MMNs and a two-dimensional topography for each condition in both groups are shown in **Figure 4**.

**Table 2:** Peak amplitudes of P100 (mean [SD], μv).

Electrode	Time widow (ms)	IGD				Control			
		Standard		Deviant		Standard		Deviant	
		Realistic	Cartoon	Realistic	Cartoon	Realistic	Cartoon	Realistic	Cartoon
PO5	90–150	3.83 (0.99)	5.28 (2.01)	3.98 (2.25)	4.92 (1.53)	1.79 (0.99)	2.19 (1.40)	2.58 (1.18)	1.92 (1.54)
PO6	90–150	2.85 (1.43)	4.47 (0.76)	2.71 (0.93)	3.96 (0.22)	3.08 (0.45)	4.34 (0.56)	3.85 (0.84)	3.97 (0.56)

IGD, Internet gaming disorder group; Control, control group; Standard, standard stimuli; Deviant, deviant stimuli; Realistic, realistic faces; Cartoon, cartoon faces.

**Table 3:** Peak amplitudes of N170 (mean [SD], μv).

electrode	Time widow (ms)	IGD				Control			
		Standard		Deviant		Standard		Deviant	
		Realistic	Cartoon	Realistic	Cartoon	Realistic	Cartoon	Realistic	Cartoon
PO5	90-150	3.83 (0.99)	5.28 (2.01)	3.98 (2.25)	4.92 (1.53)	1.79 (0.99)	2.19 (1.40)	2.58 (1.18)	1.92 (1.54)
PO6	90-150	2.85 (1.43)	4.47 (0.76)	2.71 (0.93)	3.96 (0.22)	3.08 (0.45)	4.34 (0.56)	3.85 (0.84)	3.97 (0.56)

IGD, Internet gaming disorder group; Control, control group; Standard, standard stimuli; Deviant, deviant stimuli; Realistic, realistic faces; Cartoon, cartoon faces.

**Table 4:** Peak amplitudes of P200 (mean [SD], μv).

electrode	Time widow (ms)	IGD				Control			
		Standard		Deviant		Standard		Deviant	
		Real	Cartoon	Real	Cartoon	Real	Cartoon	Real	Cartoon
PO5	210-270	1.80 (1.67)	2.70 (1.86)	1.14 (1.84)	0.27 (1.55)	2.76 (1.57)	2.82 (1.95)	1.84 (1.16)	1.41 (0.78)
PO6	210-270	2.86 (1.43)	3.83 (3.19)	1.39 (2.77)	1.37 (1.02)	2.35 (0.99)	2.96 (1.23)	1.66 (1.11)	1.34 (0.82)

IGD, Internet gaming disorder group; Control, control group; Standard, standard stimuli; Deviant, deviant stimuli; Realistic, realistic faces; Cartoon, cartoon faces.

**Table 5:** Amplitudes of mismatch negativity (MMN) (mean [SD], μv).

electrode	Time widow(ms)	IGD		Control	
		Realistic	Cartoon	Realistic	Cartoon
PO5	300–400	1.13 (2.68)	−1.33 (1.46)	0.97 (1.05)	−0.22 (1.41)
	400–500	1.41 (2.19)	−1.73 (1.43)	1.11 (1.34)	0.72 (1.21)
PO6	300–400	0.22 (2.62)	−1.56 (1.29)	0.60 (1.59)	−0.22 (0.67)
	400–500	0.75 (2.12)	−1.81 (1.53)	0.92 (1.54)	0.73 (0.56)

IGD, Internet gaming disorder group; Control, control group; Realistic, realistic faces; Cartoon, cartoon faces.

#### P100

Under the standard stimuli condition, the three-way interaction of group × face type × hemisphere was significant (*F_(1,28)_* = 14.67, *p* < 0.01, *η^2^* = 0.34). Further analysis revealed that the between-group difference in the P100 peak amplitude of the two types of faces was only significant in the left hemisphere, as reflected by the significantly larger P100 peak amplitude of both types of faces in the IGD group compared with that in the control group. The P100 peak amplitudes of cartoon faces in the IGD and control groups were 5.28 ± 2.01 and 2.19 ± 1.40, respectively (*F_(1,28)_* = 23.84, *p* < 0.01, *η^2^* = 0.46). The P100 peak amplitudes of realistic faces in the IGD and control groups were 3.83 ± 0.99 and 1.79 ± 0.99, respectively (*F_(1,28)_* = 31.49, *p* < 0.01, *η^2^* = 0.53). For the IGD group, within-group difference between the P100 peak amplitudes of the two types of faces was only significant in the left hemisphere, as reflected in the larger P100 peak amplitude elicited by cartoon faces (5.28 ± 2.01) than by realistic faces (3.83 ± 0.99; *F_(1,28)_* = 25.76, *p* < 0.01, *η^2^* = 0.48). For the control group, no significant difference was found in the P100 peak amplitude between the realistic and cartoon faces, neither in the left (*F_(1,28)_* = 1.99, *p* = 0.17) nor right hemisphere (*F_(1,28)_* = 0.89, *p* = 0.35).

Under the deviant stimuli condition, the three-way interaction of group × face type × hemisphere was not significant (*F_(1,28)_* = 0.91, *p* = 0.35). However, the two-way interaction of group × face type was significant (*F_(1,28)_* = 20.35, *p* < 0.05, *η^2^* = 0.42). A simple effect analysis revealed that cartoon faces elicited larger P100 peak amplitude in the IGD group (4.44 ± 0.73) than in the control group (2.95 ± 0.48; *F_(1,28)_* = 43.71, *p* < 0.01, *η^2^* = 0.61). Nevertheless, realistic faces did not elicit a significant difference in the P100 peak amplitude between the two groups (*F_(1,28)_* = 0.12, *p* = 0.73). Cartoon faces (4.44 ± 0.73) elicited larger P100 peak amplitude than realistic faces (3.35 ± 1.32) in the IGD group (*F_(1,28)_* = 26.32, *p* < 0.01, *η^2^* = 0.48). This significant difference did not exist in the control group (*F_(1,28)_* = 1.56, *p* = 0.22).

#### N170

Under the standard stimuli condition, the three-way interaction of group × face type × hemisphere was significant (*F_(1,28)_* = 19.25, *p* < 0.01, *η^2^* = 0.41). Further analysis revealed that in the IGD group, cartoon faces (−2.24 ± 2.17) elicited larger N170 peak amplitudes than realistic faces (−1.41 ± 1.42) in the right hemisphere (*F_(1,28)_* = 10.10, *p* < 0.01, *η^2^* = 0.27). In the control group, cartoon faces elicited larger N170 peak amplitudes than realistic faces both in the left and right hemisphere, and the N170 peak amplitudes of the cartoon and realistic faces were −2.10 ± 1.83 and −1.48 ± 1.59 in the left hemisphere, respectively (*F_(1,28)_* = 4.69, *p* < 0.05, *η^2^* = 0.14). The peak amplitudes of the cartoon and realistic faces were −2.56 ± 1.71 and −1.59 ± 1.33 in the right hemisphere, respectively (*F_(1,28)_* = 14.54, *p* < 0.01, *η^2^* = 0.34).

Under the deviant stimuli condition, no significant effects were found for N170 peak amplitude.

#### P200

Under the standard stimuli condition, the three-way interaction of group × face type × hemisphere was significant (*F_(1,28)_* = 4.37, *p* < 0.05, *η^2^* = 0.14). Further analysis revealed that, for the IGD group, realistic faces elicited significant larger P200 peak amplitudes in the right hemisphere (2.86 ± 1.42) than in the left hemisphere (1.80 ± 1.67) (*F_(1,28)_* = 7.95, *p* < 0.01, *η^2^* = 0.22), and cartoon faces elicited significant larger P200 peak amplitudes in the right hemisphere (3.83 ± 3.19) than in the left hemisphere (2.70 ± 1.86) (*F_(1,28)_* = 7.10, *p* < 0.05, *η^2^* = 0.20). For the IGD group, cartoon faces (left 2.70 ± 1.87, right 3.83 ± 3.19) elicited larger P200 peak amplitudes than realistic faces (left 1.80 ± 1.67, right 2.86 ± 1.42) in the left and right hemispheres (left *F_(1,28)_* = 5.23, *p* < 0.05, *η^2^* = 0.16, right *F_(1,28)_* = 5.44, *p* < 0.05, *η^2^* = 0.16). There was no other significant interaction effect.

Under the deviant stimuli condition, no significant interaction effects were found for P200 peak amplitude.

#### MMN

At the time range of 300–400 ms, no significant interaction effects for MMN amplitude was observed. The main effect of facial type was significant (*F_(1,28)_* = 18.61, *p* < 0.01, *η^2^* = 0.40). The MMN elicited by cartoon faces (−0.83 ± 0.21) was significantly greater than that elicited by realistic faces (0.73 ± 0.38). The main effect of hemispheres was significant (*F_(1,28)_* = 23.89, *p* < 0.01, *η^2^* = 0.46). The MMN in the right hemisphere (−0.24 ± 0.27) was significantly greater than that in the left hemisphere (0.14 ± 0.23).

During the time range of 400–500 ms, the three-way interaction of group × face type × hemisphere was not significant (*F_(1,28)_* = 0.84, *p* = 0.37). However, the two-way interaction of group × face type was significant (*F_(1,28)_* = 12.72, *p* < 0.01, *η^2^* = 0.31). A simple effect analysis revealed that cartoon faces elicited larger MMN amplitude in the IGD group (−1.77 ± 1.41) than that in the control group (0.73 ± 0.88; *F_(1,28)_* = 33.83, *p* < 0.01, *η^2^* = 0.55). Nonetheless, realistic faces did not elicit a significant difference in the MMN amplitude between the two groups (*F_(1,28)_* = 0.01, *p* = 0.93). Moreover, cartoon faces (−1.77 ± 1.41) elicited larger MMN amplitude than realistic faces (1.08 ± 2.41) in the IGD group (*F_(1,28)_* = 31.56, *p* < 0.01, *η^2^* = 0.53). No significant difference was observed between the realistic and cartoon faces in the control group (*F_(1,28)_* = 0.33, *p* = 0.57).

Because there was no significant interaction effect related to hemispheres, the average amplitudes of the left and right hemispheres were taken to do three-factor ANOVA analysis of time window 2 (300–400 ms, 400–500 ms) × type 2 (IGD group, control group) × face type 2 (cartoon, realistic). Results showed that the three-way interaction was significant (F*_(1,28)_* = 41.35, p < 0.001, η2 = 0.60). Further analysis revealed that, for the IGD group, cartoon faces (−1.44 ± 1.27 in 300–400 ms, −1.77 ± 1.41 in 400−500 ms) elicited significant larger MMN than realistic faces (0.68 ± 2.65 in 300−400 ms, 1.08 ± 2.14 in 400−500 ms) in both 300 to 400 ms and 400 to 500 ms (F*_(1,28)_* = 17.15, p < 0.001, *η^2^* = 0.38 in 300−400 ms, *F_(1,28)_* = 31.56, p < 0.001, *η^2^* = 0.53 in 400−500 ms). And they showed significant enhanced MMN in 400−500 ms (−1.77 ± 1.41) than in 300−400 ms (−1.44 ± 1.27) elicited by cartoon faces (*F_(1,28)_* = 4.31, p < 0.05, *η^2^* = 0.13), but showed significant enhanced MMN in 300−400 ms (−0.22 ± 1.01) than in 400−500 ms (0.73 ± 0.88) elicited by realistic faces (*F_(1,28)_* = 35.88, p < 0.001, *η^2^* = 0.56). For the control group, cartoon faces elicited significant larger MMN in 300−400 ms (−0.22 ± 1.01) than in 400−500 ms (0.73 ± 0.88) (*F_(1,28)_* = 35.88, p < 0.001, *η^2^* = 0.56). In both 300 to 400 ms and 400 to 500 ms, cartoon faces elicited significant larger MMN in the IGD group (−1.44 ± 1.27 in 300−400 ms, −1.77 ± 1.41 in 400−500 ms) than in the control group (−0.22 ± 1.01 in 300−400 ms, 0.73 ± 0.88 in 400−500 ms) (*F_(1,28)_* = 8.56, p < 0.01, *η^2^* = 0.23 in 300−400 ms, *F_(1,28)_* = 33.83, p < 0.001, *η^2^* = 0.55 in 400−500 ms). Other effects were not significant.

The correlation analysis between the average amplitude of the left and right hemispheres of MMN and IAT score showed that there was a significant correlation between the MMN elicited by cartoon faces and IAT scores in both 300 to 400 ms (r = −0.49, p < 0.01) and 400 to 500 ms (r = −0.74, p < 0.001), while the correlation between the realistic face MMN and the IAT scores was not significant. Also, the cartoon face MMN was significantly correlated with the IGAS scores in both 300−400 ms (r = −0.50, p < 0.01) and 400−500 ms (r = −0.72, p < 0.001), while there was no significant correlation between the realistic MMN and the IGAS scores.

## Discussion

Using the reversed deviant-standard oddball paradigm, we investigated automatic processing of individuals with IGD with regard to realistic and cartoon faces. The behavioral results showed that all the participants followed the instructions before the experiment well. According to the previous studies, this guaranteed that they are unconscious of the changes in the features of facial images during the blocks, and all the facial processing discussed in our study were automatic processing. The following findings were obtained: First, with respect to the P100 component that reflects the processing of automatic perception, in the standard and deviant stimulus conditions, cartoon faces elicited larger P100 peak amplitude in the IGD group than in the control group, and cartoon faces elicited larger P100 peak amplitude than realistic faces only in the IGD group. Second, with regard to the N170 component that reflects face-specific processing, for the IGD group, cartoon faces elicited a larger N170 peak amplitude than realistic faces in the right hemisphere, but this effect was absent in the left hemisphere. However, for the control group, this effect was present in the left and right hemispheres. Third, as far as the P200 component reflecting the processing of face configuration was concerned, the peak value of cartoon face P200 in both left and right hemispheres of IGD group was significantly greater than that of the real face. Finally, with respect to the MMN component that reflects automatic detection of facial features, cartoon faces elicited larger MMN amplitude in the IGD group than in the control group. Importantly, only in the IGD group did the cartoon faces elicit larger MMN amplitude than realistic faces.

The first finding was consistent with our hypothesis. The increased P100 peak amplitude in individuals with IGD in response to cartoon faces suggests that the ability of face processing of these individuals has been abnormal in early perceptual stage of visual processing. This result is in accordance with the findings about substance addiction and mental disorder. For example, Maurage et al. (42) found that alcoholics' brain function is impaired due to their special drinking patterns, which cause abnormalities in the P100 component that reflects early face processing. Several studies on schizophrenia have suggested that patients have abnormalities in processing faces (43, 44). Given that P100 reflects the early automatic perception of visual stimuli in the cerebral cortex (45), our results showed that in the automatic perceptual stage of visual processing, individuals with IGD exhibited an advantage effect in the automatic perception of cartoon faces.

Some researchers believe that the formation of this advantage effect may have been caused by the increased sensitivity of addicts due to their long-term exposure to addiction-related cues (46). In addition, the early visual components induced by addiction-related cues are larger than those induced by neutral cues in the addiction group (47–49). In the current study, individuals with IGD were addicted to Internet games for a relatively long time, and the characters of Internet games were all displayed with a cartoon face so cartoon faces became their addiction-related cues. Like other addicts, individuals with IGD in our study consequently showed higher sensitivity to cartoon faces than to realistic faces, resulting in an increased response to cartoon faces in automatic perceptive processing. To some extent, this kind of increasing of sensitivity may be consistent with the suppose of the incentive-sensitization model (29).

The second finding of our study was not completely consistent with our hypothesis. Although the N170 peak amplitude of individuals with IGD with regard to cartoon faces was significantly larger than realistic faces in the right hemisphere in the standard stimuli conditions, this effect was absent in the left hemisphere. However, increased N170 peak amplitude with respect to cartoon faces was observed in the left and right hemispheres of the controls. N170 is a face-selective component in the early perceptual information processing level (50). Therefore, our results show that non-addicts are significantly better at recognizing realistic and cartoon faces than IGD group. In other words, the ability to distinguish between the two types of faces was stronger in controls than individuals with IGD. He et al. (16) provided indirect support for this result. They found that the difference between the N170 peak amplitude induced by realistic faces and non-faces in the IGD group was smaller than that in the control group. Their explanation for this result was that individuals with IGD were less likely to engage in realistic social activities because of their long-term addiction to Internet games, and their sensitivity to realistic social cues (i.e., realistic faces) decreased, and their ability to distinguish between faces and non-faces was impaired. Maher et al. (51) also provided a vantage point for our result. Maher et al. (51) found no difference in the N170 amplitude between faces and non-faces for schizophrenics, and they postulated that the face-specific processing of schizophrenics may have been impaired.

In view of the above two studies and the fact that the DSM-5 and ICD-11 list IGD as a psychiatric disorder to be studied, we speculate that the early face processing of individuals with IGD may have reached the level of mental impairment, leading to the convergence of their face sensitivities to realistic and cartoon faces in the left hemisphere. Meanwhile, some researchers found that the amplitude of N170 was significantly correlated with the scores of social scales (52), and suggested that the decrease of N170 might be related to the impairment of social function (53). It also implied that the clinical manifestations of IGD individuals' difficulty in normal communication with others may be related to their abnormal early visual perception of faces.

In addition, the results of N170 were consistent with previous studies (50). There was a right hemisphere dominance effect, i.e. IGD group only showed different face detection responses to cartoon and realistic faces in the right hemisphere, while the left hemisphere could not distinguish the differences between the two types of faces. Previous studies on face processing showed that individuals have an advantage of left visual field detection, which is characterized by a more negative trend of N170 in the right hemisphere (54). Our study may illustrate that the impairment of face selection processing in IGD group also starts from the left hemisphere with weaker facial detection ability.

The third finding of our study was partially consistent with our hypothesis. Compared with realistic faces, individuals with IGD exhibit a significantly larger P200 peak amplitude elicited by cartoon faces than that elicited by realistic faces. But there was no significant difference in P200 between the groups. The results showed that, for IGD individuals, there were differences in configuration information between cartoon and real faces. Some researchers pointed out that P200 in face processing might be related to the analysis of visual characteristics of the face (55, 56). It was hypothesized that the P200 response is a perceptual matching process, which compares the input sensory information with the internal representation or memory expectation (57, 58). For IGD individuals, cartoon faces and addictive behavior have been linked, leading to cognitive salience. Our results may imply that the memory representation of individuals with IGD replace normal social faces by the cartoon faces that often appear in games. And in daily social activities, they are likely to subconsciously expect only cartoon faces. While for healthy individuals, cartoon faces are only a normal type of faces, so it was not different from realistic faces in P200. In addition, there was no significant difference in the P200 amplitude between the two groups, which may indicate that the two types of faces showed no significant difference in facial familiarity (59, 60), and the participants all successfully perceived and processed the configuration features of faces.

The final finding was consistent with our hypothesis. With respect to IGD subjects, cartoon faces elicited larger MMN amplitude than realistic faces, and cartoon faces also elicited larger MMN amplitude in subjects with IGD than in controls. These results suggested that individuals with IGD also exhibit abnormalities when it comes to detecting facial features. This finding was consistent with the results of Wu et al. (61), who revealed that patients with depression showed both abnormal processing of facial expression and a cognitive processing bias toward negative expression. Visual MMN reflects an individual's automatic detection of changes in stimulus features (61). The more individuals with IGD are sensitive to cartoon faces, the higher is their degree of automatic processing of cartoon faces. Therefore, our result indicated that individuals with IGD have the advantage of automatic detection in the features of cartoon face.

He et al. (62) used the reversed deviant-standard oddball paradigm to investigate the automatic detection advantage of Internet addicts (IAs) toward Internet-related pictures and found that Internet-related pictures elicited larger MMN amplitude in the IA group than that in the control group. They also found that Internet-related pictures elicited larger MMN amplitude than the neutral pictures in the IA group. The incentive-sensitization model was used to explain the results (29, 63, 64). This model was originally used to explain the attention bias of substance addicts toward addiction-related cues. The basic idea is that the long-term use of addictive drugs alters the function of the brain reward system associated with the addicts' nucleus accumbens, and the system gradually becomes more sensitive to drug effects and drug-related stimuli, thereby forming neural sensitization to drugs and drug-related cues (65). Functional magnetic resonance imaging studies have shown that the brain reward system of individuals with IGD exhibits an increased degree of activation in response to addiction-related cues (66–69). Following the incentive-sensitization model, He et al. (62) believed that long-term Internet addiction behavior changes the function of IAs' brain system related to the reward circuit so that the reward circuit gradually shows increased sensitivity to Internet-related cues. This neural sensitization in turn leads to Internet-related cues becoming a salient incentive. Once the incentive cues appear, addicts' reward system is automatically and quickly activated, which causes the addicts to show both a pre-attention bias toward such cues and an advantage of automatic detection toward the features of these cues.

In this sense, the incentive-sensitization model could also explain our fourth finding. For individuals with IGD, cartoon faces are addiction-related cues. Therefore, when these cues are presented to individuals with IGD, the cues automatically activate the reward system their brains and trigger their craving for Internet game due to their incentive salience. Given that cartoon faces have become the focus of cognition for individuals with IGD, the individuals display an advantage effect in automatic detection of cartoon features.

As for the time window of MMN, on the one hand, it is consistent with some researches of visual MMN (vMMN) in mental diseases that vMMN was displayed in late time windows (52). For example, Cléry et al. (70) found abnormal vMMN in Occipito-parieto-temporal sites during 300 to 410 ms in autistic children, while in another adult study of autism, they found abnormal vMMNs during 350 to 600 ms (71). Similarly, Tales and Butler (72), who studied abnormal vMMN in people with Alzheimer's disease, also found that the patients exhibited enhanced vMMN in the Occipito-temporal region over a late period (> 300 ms) than the controls, highlighting impaired automatic feature detection. This may indicate that IGD have similar neurological impairment symptoms with other psychiatric diseases, which provides neurological evidence for IGD being defined a new mental disorder.

On the other hand, the average amplitude of MMN shows significant difference between two time windows. Cartoon faces, in the IGD group, elicited significantly enhanced MMN in 400 to 500 ms than in 300 to 400 ms, while in the control group, the trend went conversely. This was consistent with the late time window of abnormal MMN in IGD subjects. That is to say, during the relatively late MMN appearance, the longer the processing time, the larger the MMN amplitude. Visual MMN has typically been observed in response to infrequent deviations in a task-irrelevant oddball sequence (73). And because of the salience of addictive cues, IGD individuals tend to show greater MMN on online game cues (63, 74). Compared with the control group, IGD subjects showed an upward trend in the later processing of MMN, which may indicate that they have difficulties to separate from the automatic processing of addiction cues (10).

Finally, our study suggested that the average amplitude of MMN was negatively correlated with the scores of the IAT and IGAS scales. Previous studies also found that there was a significant correlation between MMN and some behavioral performance scores (75) and symptoms of schizophrenia (76), which might indicate that IGD did share some cognitive neurological characteristics with certain mental disorders. Accordingly, researchers attempted to use MMN as a diagnostic indicator in clinical practice (77), such as assisting in diagnosis of schizophrenia (78), which implied that MMN might also be one of the clinical diagnostic indicators of IGD.

The limitation of the present study is that we did not quantify the social interaction function of individuals with IGD and did not analyze the correlation between the four ERP components of face processing (i.e., P100, N170, P200 and MMN) and the indicators of social function of individuals with IGD, which would have potentially affected the accuracy in the application of these four components to clinical diagnosis and the determination of symptom severity of individuals with IGD. To enhance the theoretical and applied value of research in this field, we consider not only remedying this limitation in our future research but also using facial expression as an independent variable to further explore the automatic detection of facial expression features of individuals with IGD and its relationship with indicators of social impairment (79).

## Conclusions

The present study comprehensively investigated the neurocognitive mechanism of individuals with IGD in processing realistic and cartoon faces, and found that individuals with IGD had significant abnormalities or impaired functions in the early automatic perception of faces, face-specific processing, face configuration processing, and the detection of facial features. In the initial stage of visual perception, individuals with IGD showed an enhanced advantage in automatic perception of cartoon faces. In the subsequent stages of face-specific and face configuration processing, individuals with IGD had difficulty distinguishing between cartoon faces and realistic faces. Finally, in the stage of automatic detection of facial features, individuals with IGD exhibited an advantage in automatic detection of cartoon face features. The theoretical significance of our study was to aid in the understanding of the mechanism of IGD from the neurocognitive perspective of facial recognition, and the practical value was to find objective ERP indicators for the clinical diagnosis of IGD.

## Data Availability Statement

The raw data supporting the conclusions of this manuscript will be made available by the authors, without undue reservation, to any qualified researcher.

## Ethics Statement

The studies involving human participants were reviewed and approved by the Ethics Committee of the School of Psychology of Central China Normal University. The patients/participants provided their written informed consent to participate in this study.

## Author Contributions

JH and LF designed and finished the experiment, and dealed with all data. YZ joined in the design of the experiment. LF, YZ, TP, and YN co-wrote the manuscript. YZ organized and revised the manuscript. All co-authors checked and approved the manuscript.

## Funding

This study was funded by the Fundamental Research Funds for the Central Universities (CCNU19Z02009) and the National Natural Science Foundation of China (31571139).

## Conflict of Interest

The authors declare that the research was conducted in the absence of any commercial or financial relationships that could be construed as a potential conflict of interest.
